# Vascular flow reserve as a link between long‐term blood pressure level and physical performance capacity in mammals

**DOI:** 10.14814/phy2.12813

**Published:** 2016-06-02

**Authors:** Christian B. Poulsen, Mads Damkjær, Bjørn O. Hald, Tobias Wang, Niels‐Henrik Holstein‐Rathlou, Jens Christian B. Jacobsen

**Affiliations:** ^1^ Department of CardiologyAarhus University HospitalAarhus NDenmark; ^2^ Department of Clinical MedicineAarhus UniversityAarhus NDenmark; ^3^ Hans Christian Andersen Children's HospitalOdense University HospitalOdense CDenmark; ^4^ Department of Biomedical SciencesUniversity of CopenhagenCopenhagenDenmark; ^5^ Department of ZoophysiologyAarhus UniversityAarhusDenmark

**Keywords:** Blood pressure, endothelial function, flow reserve, microcirculation, model, resistance

## Abstract

Mean arterial pressure (MAP) is surprisingly similar across different species of mammals, and it is, in general, not known which factors determine the arterial pressure level. Mammals often have a pronounced capacity for sustained physical performance. This capacity depends on the vasculature having a flow reserve that comes into play as tissue metabolism increases. We hypothesize that microvascular properties allowing for a large vascular flow reserve is linked to the level of the arterial pressure.To study the interaction between network properties and network inlet pressure, we developed a generic and parsimonious computational model of a bifurcating microvascular network where diameter and growth of each vessel evolves in response to changes in biomechanical stresses. During a simulation, the network develops well‐defined arterial and venous vessel characteristics. A change in endothelial function producing a high precapillary resistance and thus a high vascular flow reserve is associated with an increase in network inlet pressure. Assuming that network properties are independent of body mass, and that inlet pressure of the microvascular network is a proxy for arterial pressure, the study provides a conceptual explanation of why high performing animals tend to have a high MAP.

## Introduction

The circulatory system is able to deliver a flow that matches the demand of every tissue in the body. This requires that the mean arterial blood pressure (MAP) is sufficient to drive an adequate blood flow through the resistance imposed by the vasculature, particularly the microcirculation. Despite differences in body size, metabolism, and natural habitat, MAP is remarkably constant across mammals (Seymour and Blaylock 2000). Mammals are typically also “high performers” capable of sustained physical activity. This raises a question as to the presence and nature of a general link between performance capacity and blood pressure level.

The presence of a basal tone in the resting state underlies the ability of the resistance vasculature to deliver a massive increase in flow in response to increased demand, as seen in, for example, skeletal muscle tissue at the onset of physical exercise (Saltin et al. 1998). This vascular flow reserve (VFR), that is, maximal flow relative to resting state flow (Jacobsen et al. 2010), is an evident priority of the circulatory system (Hoffman 1984; Pedrinelli et al. 1994). A functional VFR also depends on the ability of the heart to react according to the Starling mechanism, continuously passing on exactly the amount of blood it receives (Shiels and White 2008). Since the VFR depends on microvascular resistance and since the latter is a main determinant of arterial blood pressure, we hypothesize that the functional VFR properties of the microcirculation are linked to the arterial pressure level in mammals.

To address this hypothesis, we developed a parsimonious model of a bifurcating microvascular network where noncapillary vessels can adapt structurally as regards both lumen diameter and wall thickness. We assume that inlet pressure of the network can serve as a proxy for arterial pressure because the pressure drop across the microcirculation is large compared to that along conductance vessels. Hence, microvascular resistance will, everything else being equal, be reflected in the systemic arterial pressure level.

During simulations of remodeling and growth, capillaries were constrained to maintain a flow that matched the tissue demand, whereas noncapillary vessels were required to maintain stress homeostasis of their walls. Adaptation of network structure and inlet pressure was investigated in response to changes in (1) endothelial influence on vessel wall tone and (2) the level of basic vessel wall tone. In each case, while requiring the same network flow, parameter regions that gave rise to high precapillary resistance and hence a high VFR, were associated with a high network inlet pressure. This indicates that a high VFR in high performing animals necessitates a high arterial pressure level.

## The Model

A qualitative description of the model follows below. Key variables and constants are summarized in the Table 1. A full model description is provided in the Appendix.

**Table 1:** Glossary**:** *Var*: a variable in the model. *Con*. A parameter which is constant in the individual vessel in a given simulation but which may vary between simulations

Variable (var)/constant (con)	Description and unit Pascal (Pa), meters (m), seconds (sec)
Mean arterial pressure *(MAP)*	Mean arterial pressure (Pa) (approx. diastolic pressure + 1/3 pulse‐pressure)
Vascular flow reserve *(VFR)*	Vascular flow reserve (flow during maximal vasodilatation relative to resting‐state flow)
*P*_in_(v*ar*)	Network inlet pressure (Pa)
*P*_out_(c*on*)	Network outlet pressure (Pa)
*P*_node_(v*ar*)	Pressure in a node of the network (Pa)
$\overset{¯}{P}$	Average transmural vessel pressure (Pa)
*ΔP*(*var*)	Pressure decline along a vessel (Pa)
*ψ*(v*ar*)	Instantaneous activation of the contractile apparatus (no unit)
*ψ*_*pss*(v*ar*)_	Pseudo steady state activation of contractile apparatus (no unit)
*ψ*_habitual(con)_	Long‐term equilibrium level of activation (no unit)
$\overset{¯}{S}(\mathrm{var})$	Circumferential stress averaged over the layers of the wall (Pa)
${\overset{¯}{S}}_{\mathrm{habitual}}\left(\mathrm{var}\right)$	Long‐term equilibrium level of $\overset{¯}{S}$ (Pa)
*τ*(var)	Wall shear stress (Pa)
*γ*(con)	Sensitivity to wall shear stress (no unit)
*C*_wall_(v*ar*)	Shear‐stress influence on the wall
*c*_wall,habitual_(c*on*)	Long‐term equilibrium level of shear‐stress influence on the wall
*v*(con)	Capillary flow‐velocity (m/sec)
*m* (var)	Metabolic factor (no unit)
*Q* (var)	Flow (m^3^/sec)
$\begin{array}{cc} & t_{\mathrm{activation}}\;\left(con\right),\;t_{\mathrm{remodelling}}\;\left(con\right),\;\\ & t_{\mathrm{trophic}}\;\left(con\right) \end{array}$	Time constant for the indexed process (sec)

Structural variable (var)/Structural constant (con)	
r_*i*_ (var)	Inner vessel radius in the active, pressurized vessel (m)
*ρ*_*i*_(*var*)	Inner radius (“structural inner radius”) of fully relaxed vessel at 0 Pa transmural pressure (m)
*η* (var)	Relative wall‐thickness (outer radius divided by inner, no unit). Fully relaxed vessel at 0 Pa transmural pressure
*l* (con)	Vessel length (m)

### Vessel wall model

The vessel wall consists of passive elastic material (e.g., collagen and elastin) arranged in parallel with an active contractile component. As the wall is distended, the stress contribution from the elastic material increases in an exponential fashion. In contrast, the contribution from the active contractile part, (i.e., the SMC, smooth muscle cell), has a triangular shape, that is, distension beyond a certain point (forced dilatation) causes a decline in active stress (Feldberg et al. 1995; Jacobsen et al. 2008) (Appendix A).

### Activation of the smooth muscle cell contractile machinery

**Figure 1:**
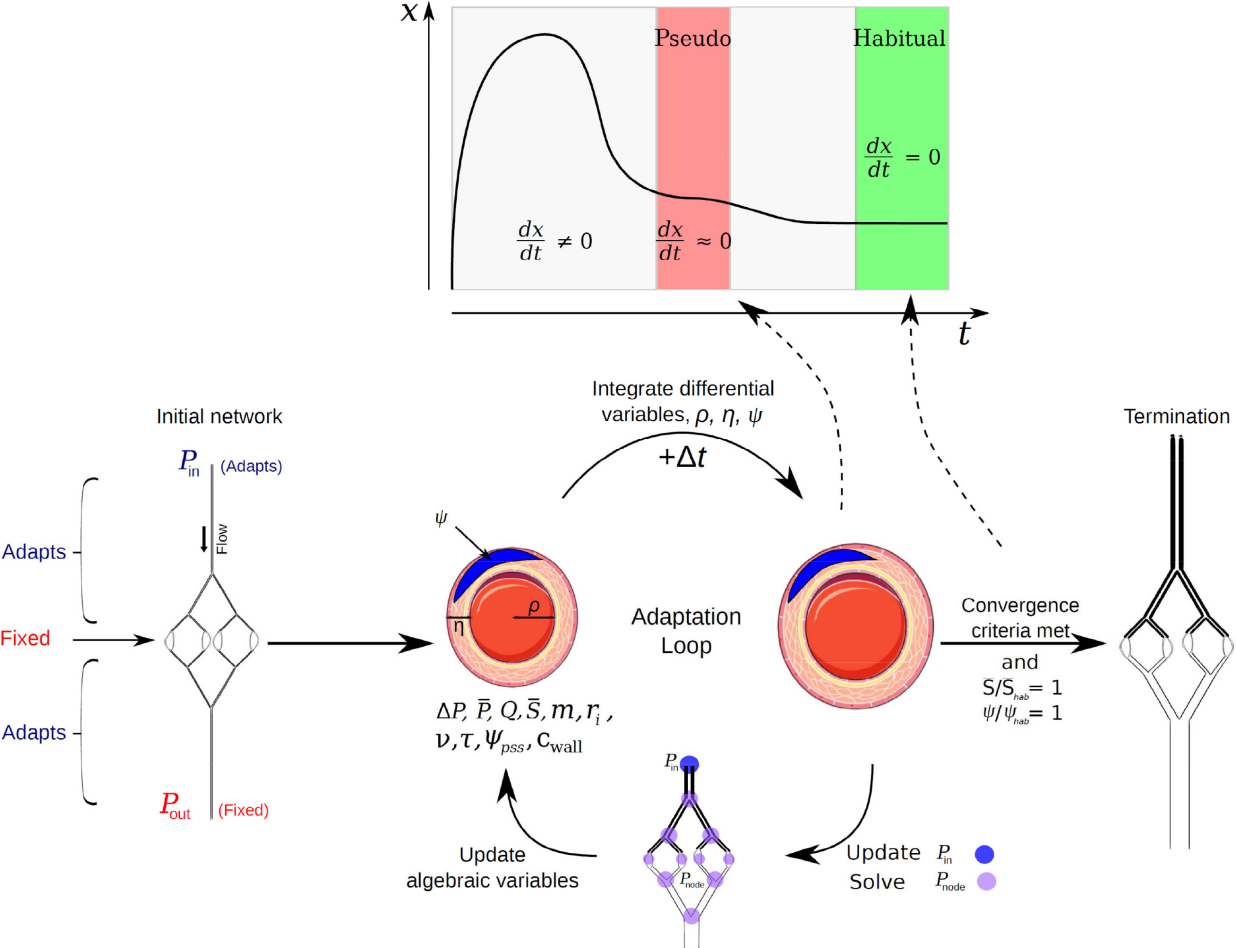
Upper part: Illustration of the difference between pseudo steady state where only fast transients have decayed and the true steady state (identical to the habitual state of the vessel) where both fast and slow transients have decayed. Lower part: The basic iteration loop of the program. Left side shows the initial network configuration. All noncapillary vessels (vessels with adaptive potential) are initialized with the same radius and relative wall thickness. Right side shows vessel morphology after structural adaptation (schematic). Note the arterio‐venous difference in vessel radius and relative wall thickness.

The instantaneous activation, *ψ* of the SMC lies between 0 (no activation) and 1 (maximal activation). For a given state of a vessel (i.e., when having a given radius, transmural pressure and flow) the pseudo steady‐state level of activation, *ψ*_pss_, is determined by the sum of the influences from the different vasomotor mechanisms (in the present model the myogenic response and the shear‐ stress‐mediated mechanism*,* please see below). The *ψ*_pss_ represents the activation the vessel should have under the given set of inputs (Appendix B). It is termed a “pseudo steady state” because the system may still drift due to slower structural changes (i.e., remodeling and growth, cf. Fig. 1, upper part). Any perturbation (e.g., a change in transmural pressure or flow) will cause a change in *ψ*_pss_ which is followed immediately by a change in *ψ*, the instantaneous level of activation, which is driven toward, *ψ*_pss_. This process takes place on the timescale of short‐term flow‐regulation (sec to min). Thus, *ψ*_pss_ rarely represent the final state of the vessel, but rather a situation where only fast transients have decayed (Appendix B).

### Vasomotor mechanisms

The model includes three mechanisms which collectively determine SMC activation.

#### The myogenic response

The SMCs of the vessel wall are sensitive to the average level of circumferential stress, *S*. According to the law of Laplace the transmural pressure, *P* is proportional to the circumferential stress. An increase in transmural pressure will cause an increase in wall stress, which, subsequently, will lead to an the activation of the SMC. Depending on the specific properties of a given vessel, an increase in transmural pressure may therefore lead to a more or less pronounced constriction. Vessels of different size (i.e., taken from different positions in the network) have different myogenic reactivity (please see Appendix C).

#### The shear‐stress‐sensitive mechanism

The vascular endothelium influences activation of the SMCs through a variety of mechanisms. Particularly, wall shear stress, *τ*, is known to influence both SMC activation (Koller et al. 1993,1994) and wall structure (Unthank et al. 1996). The vascular wall is under a certain tonic influence from *τ*. An increase in *τ*, leads to SMC relaxation (Koller et al. 1993, 1994). We make no assumptions as to the specific equilibrium level of *τ* in a given vessel or in the networks as a whole, but rather assume that it is the influence*, c*_wall_ of the endothelium on the vascular wall that matters. This implies taking into account not only the level of *τ* influencing a given endothelial surface area with a sensitivity governed by an “endothelial function”, *γ* but also considering the underlying amount of wall material, that is, the difference between thick‐ and thin‐walled vessels (for details on this formulation please see Appendix D).

#### Response to tissue metabolism

Every part of the tissue must on average be sufficiently perfused, that is, no longstanding over‐ or under‐perfusion. It is assumed that tissue metabolic need is satisfied when blood flows through the capillary bed at a certain rate. With capillary size being invariant, this corresponds to a certain capillary flow‐velocity. In the present simple formulation a dimensionless variable, *m* carries the information of whether the actual capillary flow meets the tissue metabolic need, represented by a desired capillary flow. A positive *m* means that tissue metabolism exceeds what can be met by the flow at a given moment, that is, the tissue is under‐perfused. In that case the network perfusion pressure, *P*_in_ will increase until *m* vanishes, that is, when perfusion matches metabolism. The same process, but in the opposite direction (negative *m*), takes place in case of overperfusion. In this way *P*_in_ will eventually reach the level where flow matches tissue demand in the fully adapted network. In the model this process does not imply a specific physiological mechanism, although it has similarity to pressure driven volume regulation (Guyton 1987) (please see Appendix E and Discussion). Note that values chosen for the target capillary flow does not represent any specific tissue; it follows from anatomically and physiologically reasonably (textbook) values for capillary dimensions and flow‐velocities.

### The habitual state

The present model operates with three different habitual variables, the habitual circumferential wall stress, ${\overset{¯}{S}}_{\mathrm{habitual}}$, the habitual activation, *ψ*_habitual_, and the habitual level of endothelial influence on the wall, *c*_wall, habitual_. The habitual state represents a true steady state where all transients have decayed as opposed to the pseudo steady state where only fast transients have decayed (cf. Fig. 1, upper part). The habitual state should be thought of as a long‐term homeostatic point, at which the function of the vascular wall is optimal as regards its capacity in acute flow regulation. In particular, the resistance network must hold a basal (i.e., habitual) tone that can be enhanced or reduced by acutely acting mechanisms, for example, vascular conducted responses, to retain the possibility of regulating flow either up or down following acute changes in tissue metabolism. All noncapillary vessels will adapt structurally (i.e., by changes in lumen radius and/or in the amount of wall material) to approach this point, so that eventually:

$$\frac{\overset{¯}{S}}{{\overset{¯}{S}}_{\mathrm{habitual}}}=\frac{\psi }{\psi_{\mathrm{habitual}}}=\frac{c_{\mathrm{wall}}}{c_{\mathrm{wall},\mathrm{habitual}}}=1$$

Note that larger vessels operate at higher stress levels. To account for that, (see Appendix F for details),${\overset{¯}{S}}_{\mathrm{habitual}}$ was implemented as being a function of the structural internal radius *ρ*_*i*_. Therefore ${\overset{¯}{S}}_{\mathrm{habitual}}$ is a variable in the individual vessels changing alongside with *ρ*_*i*_.

### Remodeling and growth

We follow the standard nomenclature of vascular adaptive changes in which a structural change in luminal radius of a vessel is referred to as remodeling (inward or outward) whereas a change in the amount of vessel wall material is referred to as a trophic response (hypo‐ or hypertrophic). A remodeling response without a simultaneous change in total amount of wall material (i.e., cross‐sectional area of the wall is constant) is referred to as eutrophic (Mulvany 1999).

In accordance with experimental observations a remodeling response is caused by a sustained change in activation of the vascular wall away from its habitual, level. An increase in activation causes inward remodeling (Bakker et al. 2002; Martinez‐Lemus et al. 2004), whereas sustained relaxation of the wall causes outward remodeling (Pistea et al. 2005). In the present formulation it is therefore the difference between *ψ* and *ψ*_habitual_ at any given moment, that determines the direction (and rate) of the remodeling response. Remodeling changes the structural radius of the vessel without changing the cross‐sectional area of the vessel wall, that is, it is a eutrophic response.

In contrast, a trophic response involves a change in the amount of wall material and is driven by a sustained change in wall stress, $\overset{¯}{S}$ away from ${\overset{¯}{S}}_{\mathrm{habitual}}$. If $\overset{¯}{S}>{\overset{¯}{S}}_{\mathrm{habitual}}$ the response will be hypertrophic; in the opposite case it will be hypotrophic (see Appendix F).

### Time scales

Acute regulation of vascular tone, remodeling, and growth take place in all noncapillary vessels in the network. They occur simultaneously but are de facto separated by their different time scale of operation, as summarized in Table 2. The time scale characteristic of each process, reflects how long a given stimulus must be present to induce a change. Note that although the model is in principle dynamic, only results from the final steady state are displayed in the results section.

**Table 2:** Summary of model timescales. Modified after Jacobsen and Holstein‐Rathlou (2012) with permission

Time scale	Changes in	Function	Stimulus	Formulation
Sec/min	*ψ*	Acute regulation of lumen radius. No change in *ρ*_*i*_ or *A*	Short term deviation of *ψ* from *ψ*_pss_	$\frac{d\psi }{dt}=\frac{1}{t_{\mathrm{activation}}}\left(\psi_{\mathrm{pss}}-\psi \right)$
H/days	*ρ*_*i*_	Structural regulation of lumen radius (remodeling). No change in *A*	Intermediate term deviation of *ψ* from *ψ*_habitual_	$\frac{d\rho_{i}}{dt}=\frac{1}{t_{\mathrm{remodeling}}}\left(\psi_{\mathrm{habitual}}-\psi \right)\rho_{i}$
Weeks/months	*A*	Structural regulation of wall cross‐sectional area (trophic response)	Long term deviation of $\overset{¯}{S}$ from ${\overset{¯}{S}}_{\mathrm{habitual}}$	$\frac{d\eta }{dt}=\frac{1}{t_{\mathrm{trophic}}}\left(\overset{¯}{S}-{\overset{¯}{S}}_{\mathrm{habitual}}\right)$

### Network structure

An example of a bifurcating network similar (though smaller) to those used in the simulations is shown in Figure 1, lower part. Note that the applied networks only include small vessels from the microcirculation. Conductance vessels are not included in the model. Left side of Figure 1 shows the situation before (initial state) structural adaptation. Right side shows the situation after (final state) structural adaptation. All vessels are initiated with a certain length, the size of which depends on the vessel generation and which remain invariant throughout a simulation (Jacobsen et al. 2010). Average vessel length increases symmetrically away from the capillary bed on both sides. All capillaries are 50 *μ*m in length.

As indicated in Figure 1, network shown to the left, all noncapillary vessels (i.e., vessels with adaptive potential) are initiated with the same structural internal radius and the same relative wall thickness (Table 3). During the subsequent simulation, these two variables are allowed to adapt freely. In capillaries, however, they are kept constant. The schematic of the resulting structure (network to the right) shows that for comparable generations on the two sides of the capillary bed, arterial vessels become narrower and thick‐walled.

**Table 3:** Standard parameter setting and initial values of model variables and parameters (values used in the basis simulation of Fig. 2)

Variable (var)/constant (con)	Initial value and unit Pascal (Pa), meters (m), seconds (sec)
*P*_in_ (initial, var)	4000 (Pa)
*P*_out_(*con*)	2000 (Pa)
*ρ*_*i*_ (initial, var)	*ρ*_*i*_ = 20 × 10^−6^(m)
*η* (initial, var)	1.125 (no unit)
G(con)	4 (no unit) Generation number for terminal arterioles (cf. Fig. 2).
*v* (con)	2 × 10^−3^Capillary flow‐velocity (m/sec)
Capillary radius (con)	3 × 10^−6^(m)
*γ* (con)	0.65 (no unit)
$c_{\mathrm{wall},\mathrm{habitual}}$(con)	1.2 × 10^6^

*Var*: a variable in the model. *Con*. A parameter which is constant in the individual vessel in a given simulation but which may vary between simulations.

### Network hemodynamics

Each vessel is considered as a single segment and a node is a junction of three such vessels (a bifurcation point in the vascular tree shown in Figure 1). Assuming that no fluid is lost to the interstitium, the sum of the flows entering and leaving any node equals zero (Kirchhoff's law). On that basis a linear system can be solved to obtain node pressures (Jacobsen et al. 2003). Subsequently, all other hemodynamic and vessel wall variables can be calculated (Appendix G).

The vascular flow reserve is defined as network flow in the relaxed network (for *ψ* = 0 all noncapillary vessels) divided by the flow in the active network (for all *ψ* = *ψ*_habitual_ noncapillary vessels) with the same *P*_in_ and *P*_out_ in the two cases.

#### Program structure

Figure 1 outlines the program flow. The upper part illustrates the difference between pseudo steady state where only fast transient have decayed, and the true steady state where all transients have decayed, and each vessel in the networks has reached its habitual state. The lower part of Figure 1 illustrates how, using the initial network boundary pressures and the initial values of internal vessel radii, the node pressures are calculated, followed by calculation of start values for the algebraic variables ($\Delta P,\overset{¯}{P},Q,\overset{¯}{S},\tau ,\psi_{\mathrm{pss}},v,\;m,c_{\mathrm{wall}}$ and *r*_*i*_) for each vessel in the network.

The program now enters the basic loop shown in Figure 1. Based on the updated algebraic variables, new values are found for the differential variables *ρ*_*i*_, *η* and *ψ* (Table 1). This loop is then intersected with updating of *P*_in_ and node pressures as follows: while network outlet pressure, *P*_out_ is maintained constant, the inlet pressure, *P*_in_, starting from an arbitrary value is free to drift. If desired total capillary flow is too small (i.e., positive *m*) inlet pressure is increased. If it is too large, inlet pressure is reduced. Node pressures throughout the network are then recalculated and the loop continued with updating of algebraic variables, integration of differential variables and so forth.

The process is continued until every vessel and node in the network simultaneously fulfill the convergence criteria given below (please also see Appendix H).

#### Initial and boundary conditions

Each noncapillary vessel in the network is characterized structurally by the length, (*l*), the internal structural radius,(*ρ*_*i*_), and the relative wall thickness (*η*). Since capillaries are modeled as having no adaptive potential they remain at their starting radii throughout. Consequently, variables relating to the capillary wall have no functional role and are not calculated (breaks in Fig. 3G–I). Unless otherwise stated, initial values are those given in Table 3, but the specific choice of initial values did not change the conclusions.

Due to the computational burden only relatively small networks were used in the simulations; using larger networks in test simulations did not reveal qualitative differences and did not alter the conclusions. However, in a larger network *P*_in_ will end up being larger corresponding to a more upstream position in the circulatory system.

#### Convergence criteria

Convergence is evaluated by comparing values of specific variables between iterations separated by a certain number of rounds (Jacobsen et al. 2010). The variables $r_{i},\rho_{i},\eta ,\psi ,v,\overset{¯}{S},P_{\mathrm{node}}$(including *P*_in_), and total network flow (below all symbolized by *χ*) are evaluated under the following criterion:

$$\delta =\left|\frac{\chi_{n+5000}}{\chi_{n}}-1\right|<10^{-2}$$

where *δ* is the relative change in the value of the given variable from time‐step *t*_n_ to *t*_n+5000_. At termination this criterion must be fulfilled simultaneously for each noncapillary vessel and each node in the network. The large number of steps (here arbitrarily set to 5000) between consecutive evaluations of *δ* was chosen to save computation time. In addition it is a criterion that at termination all noncapillary vessels must be in their habitual state:

$$\delta_{\mathrm{habitual}}=\left|\frac{Z}{Z_{\mathrm{habitual}}}-1\right|<10^{-2}$$

where *Z* represents $\psi ,\overset{¯}{S}$ or *c*_wall_ (please also see Appendix I).

#### Source code

The source code was written in C (ANSI C standard) by the authors, using Microsoft Developer Studio (Visual C++ 6.0, professional ed, Microsoft, Seattle, WA). The model was integrated using the Euler method, and results were visualized in Grapher 10.0 (Golden Software Inc. Golden, CO). Simulations are performed on a Pentium III, quadcore personal computer.

## Results

### Structural network adaptation

**Figure 2:**
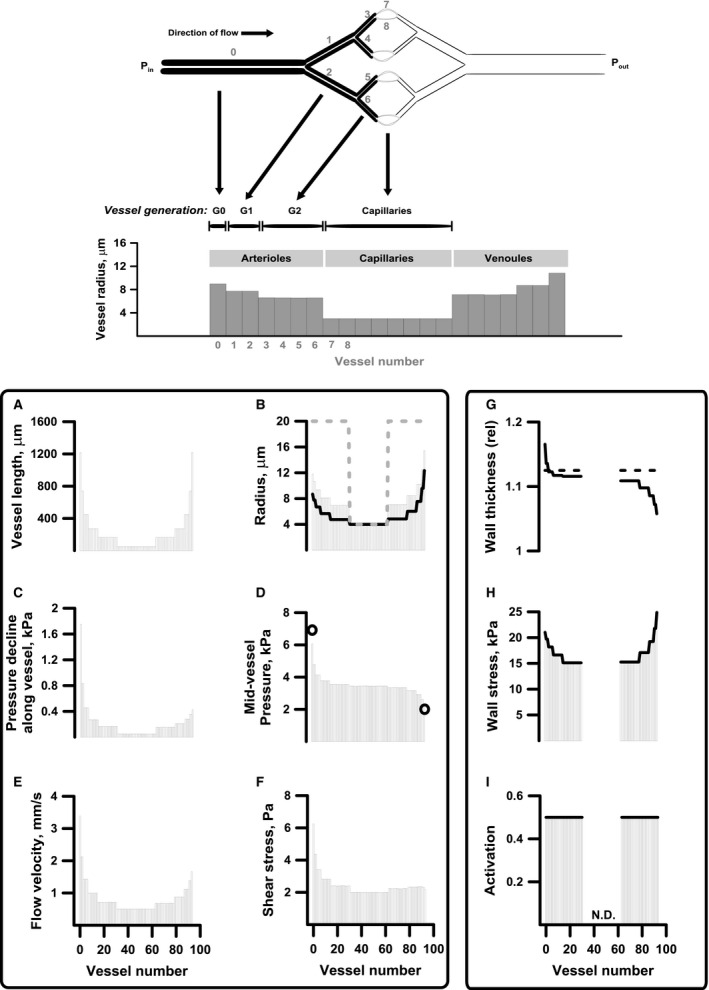
Top panel: Mapping of network properties onto a column diagram. Vessel numbers and generation (order) numbers are indicated in gray and black, respectively. Bottom panels: Basic simulation showing structural and hemodynamic constants and variables defined for all vessels in the network (Pannel A–F) and those only defined in noncapillary vessels (G–I). Please see text for details.

Figure 2, top panel illustrates how different network quantities are mapped into bar‐plots (see legend for details). The lower panels A–I shows a simulation of structural adaptation of a network with five generations of arterioles, yielding a total of 94 vessels. Panel A shows the symmetric distribution of vessel‐lengths that remain constant during a simulation. In Panel B the dashed line shows the structural internal radius (*ρ*_*i*_) before adaptation. *ρ*_*i*_ of all noncapillary vessels are initially set (arbitrarily) to five times the capillary radius. The final stable state is independent of the initial size of *ρ*_*i*_. The columns of Panel B show structural internal radius (*ρ*_*i*_) after adaptation and the full black line shows vessel radii (*r*_i_) when the network is pressurized and vessels have developed tone. Note that for comparable generations, the high‐pressure arterial side vessels become smallest. This is a consequence of inward remodeling caused by stress induced activation. The panels C and D show the pressure decline along a given vessel and the mid‐vessel pressure, respectively. The circles of panel D indicate *P*_in_ and *P*_out_. Since vessels on the arteriolar side of the capillary bed become narrow, pressure decline is largest in these vessels. In turn, this is reflected in both flow velocity and shear stress as shown in panels E and F.

The figure group G, H, and I shows variables that are not defined (N.D.) for capillary vessels in the center of the network. Panel G shows the relative wall thickness (*η*) before (dashed line) and after (full line) adaptation. Note that arterial vessels become thick‐walled compared to venous vessels. Panel H show circumferential wall stress (columns) which become larger in larger vessels and, as the simulation settles, in each vessel attain the habitual value marked by the full black line. Finally panel I shows the activation (columns). In each vessel the activation eventually reach the habitual value (full line). Note that, although the patterns would be similar, the exact values of the results shown in Figure 2 depend on the parameter setting (in particular *γ* and *ψ*_hab_)used in the specific simulation. Collectively Figure 2 shows a model behavior in accordance with that observed in vivo; development of an arterio‐venous asymmetry, stable *P*_in_ and a flow that matches the tissue demand, while the network has a flow reserve due to the presence of tone in noncapillary vessels.

### Relationship between vascular flow reserve, network inlet pressure, and endothelial function

**Figure 3:**
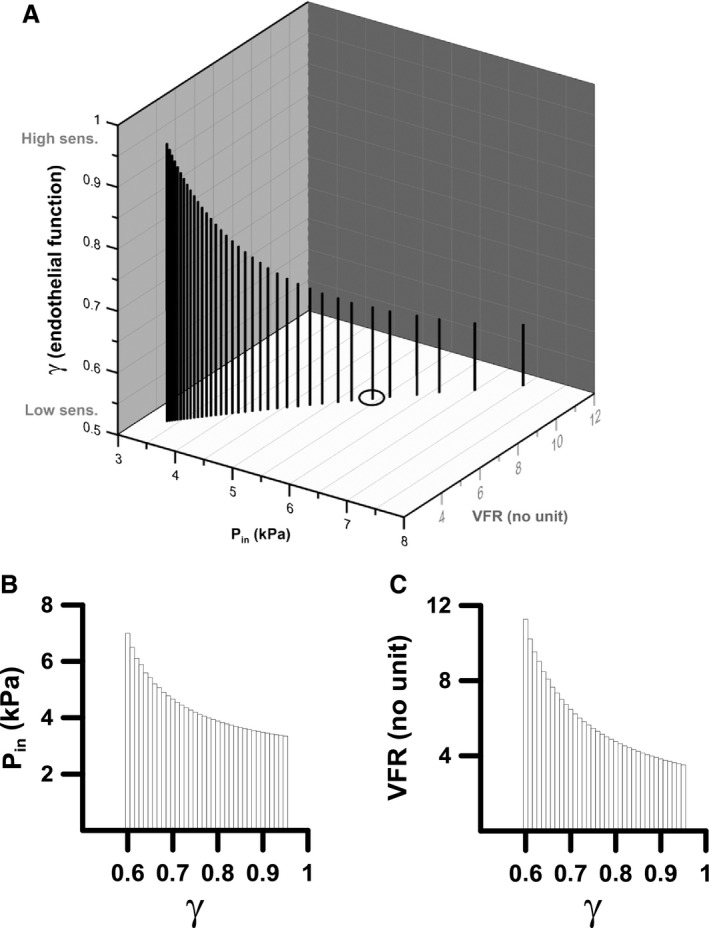
Pannel (A): *P*_in_ and vascular flow reserve (VFR) as a function of network endothelial function. Capillary radius (3 *μ*m), flow‐velocity (2 mm/sec), and habitual activation (0.5) remain invariant. Each bar represents a single simulation. For clear visualization *γ*, which is given a new value in each simulation, appears on the *y*‐axis. Obtaining a specific flow reserve (column marked by a circle), is possible through a shift in the endothelial sensitivity to shear stress, *γ* however, shifting the latter will cause a simultaneous change in network inlet pressure. Hence, a high flow reserve comes with the price of a high *P*_in_ Panels (B and C) show the same simulations as (A), but with endothelial function on the *x*‐axis.

A high value of *γ* corresponds to an endothelium that has a high sensitivity to shear stress, that is, only a low shear‐stress level is required to maintain a certain vessel diameter. In that case noncapillary vessels will become large and, as shown in Figure 3A, *P*_in_ will become low. A relatively large fraction of the total network resistance will reside in the capillary bed and not in the arterioles. Consequently, complete vaso‐relaxation will result in a modest increase in flow, corresponding to a low VFR.

In contrast, low values of *γ* means an endothelium with low sensitivity to shear stress. Noncapillary vessels will become narrow and resistance to flow will reside almost entirely in the arteriolar network. Hence, complete vasodilatation will result in a large increase in flow, that is, a large VFR. Note that in all simulations capillary radius and flow‐velocity is the same and all noncapillary vessels in all cases reach their habitual state. If *γ* become too small (on the order of 1/2), equivalent to a pronounced “endothelial dysfunction”, the network becomes unstable and enters a vicious cycle with an ever increasing *P*_in_ leading to progressive narrowing of the arterial side vessels and so forth (not shown).

Figure 3A also illustrates how requiring a specific vascular flow reserve (for instance bar marked by a ring), is fulfilled for a specific combination of endothelial function and pressure. On adaptation to circumstances that require a high VFR, a reduced *γ* will result in the desired VFR but only on the expense of a higher pressure (reaching the marked bar from the left). On the other hand, if the flow reserve is larger than required, increasing *γ* will result in a reduction in the VFR and in that case the organism can do with a less expensive lower pressure level (reaching the marked bar from the right). For clarity and using the same simulations as in panel A, the panels B and C displays the relations between VFR and *γ* as well as between *P*_in_ and *γ*.

### Vascular flow reserve and inlet pressure following network adaptation under different levels of *ψ*_habitual_

**Figure 4:**
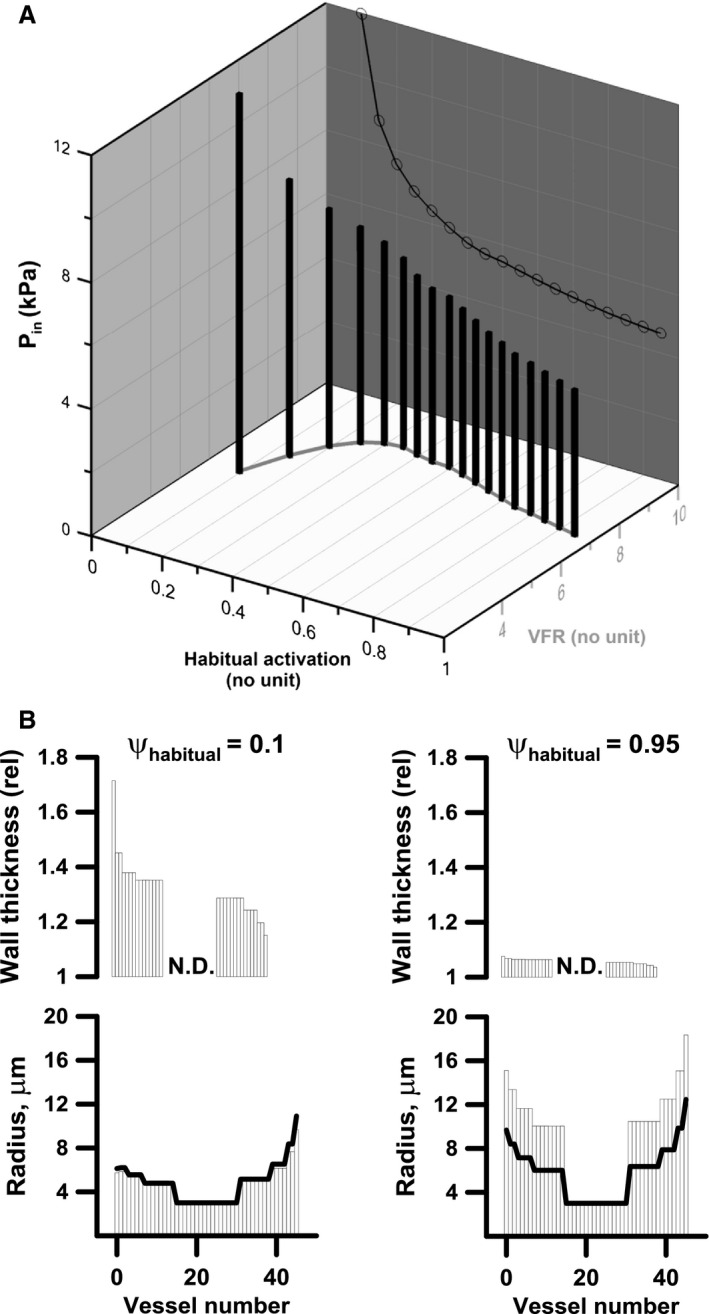
(A) Shows vascular flow reserve (VFR) and pressure for networks adapted under different levels of habitual activation. Each bar represents a single simulation. Gray line and line with open circles are projections highlighting VFR and *P*_in_ respectively, versus habitual activation. In the course of the simulation *γ* (0.65), capillary radius (3 *μ*m) and capillary flow‐velocity (2 mm/sec) remain invariant. When the level of habitual tone is low (e.g., 0.1) VFR also becomes low since vasodilatation has little effect but due to a hypertrophic response the vessels become thick‐walled (B left, upper panel, relative wall thickness, (*η*) and narrow (Fig. 4B left, lower panel. Columns: structural radius (*ρ*_*i*_), Black line: radius with tone (*r*_*i*_)). For high levels of habitual activation (e.g., 0.95) there is a decline in VFR and *P*_in_(for further explanation please see text) and the vessels become thin‐walled and large (Fig. 4B right panels).

**Figure 5:**
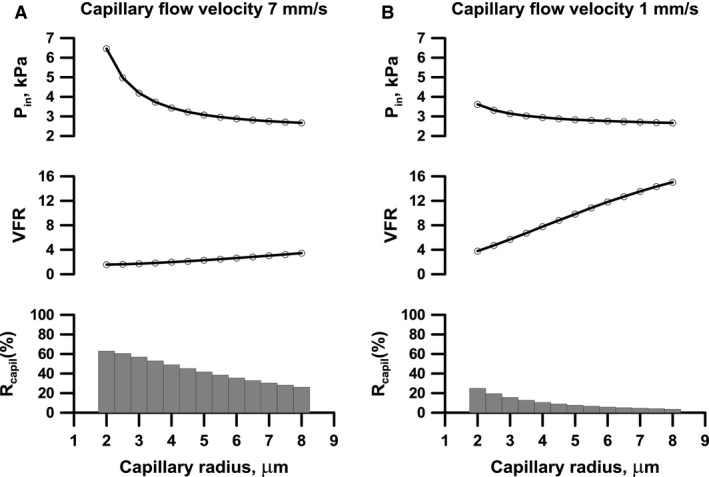
*P*_in_ VFR and resistance of the capillary bed, *R*_capil_, relative to total network resistance as a function of capillary radius at high‐ (Panel A) and low (Panel B) capillary flow velocity. The surrounding network can adapt structurally resulting in similar *P*_in_ over a range of radii and flow velocities unless capillary bed resistance is too high. The resulting VFR, however, varies substantially (see text for details).

Figure 4A shows VFR and network inlet pressure for networks adapted under different levels of *ψ*_habitual_, that is, in the final state all noncapillary vessels should have an activation equal to *ψ*_habitual_, (similar to the “basal tone” in vivo). Gray line and line with open circles are projections highlighting VFR and *P*_in_ respectively, versus habitual activation. As in Figure 3 capillary radius and flow‐velocity are the same in all simulations. When *ψ*_habitual_ is low (e.g., 0.1) VFR also becomes low since acute vasodilatation has little effect. However, low tone makes the wall material soft, that is, the increase in stress following an increase in strain is small. Consequently, as shown in Figure 4B left, the vessels become thick walled (upper panel) in order carry the transmural pressure. This hypertrophic response results in vessel narrowing (lower panel, columns are structural radius, black line is radius with tone) because it lowers the effect of shear stress (c.f. Eq. A11 in Appendix); in turn this causes an increase in inlet pressure. Somewhat counterintuitively, there is a decline in VFR at the highest levels of *ψ*_habitual_ (e.g., 0.95). In that case the wall material is rigid and a limited amount of wall material is needed to carry the transmural pressure. This increases the effect of shear stress (c.f. Eq. A11) causing the vessels to become large and thin walled (Fig. 4B, right) and pressure becomes low. When the arterial vessels are large, a larger fraction of the network resistance resides in the capillary bed (c.f. Fig. 5) reducing the effect of upstream vasodilatation and hence reducing VFR.

Vascular flow reserve is largest at intermediate values of *ψ*_habitual_ (peaking at 0.35, indicated by gray line in the *ψ*_habitual_−VRF plane). This is also the most appropriate from a point of view of efficient short‐term regulation of flow, since it allows for both up‐ and down regulation of vessel tone, that is, short‐term deviation of *ψ* from *ψ*_habitual._

### Design and properties of the capillary bed – effect of variation in capillary radius and flow velocity

Figure 5 shows *P*_in_, VFR and resistance of the capillary bed, *R*_capil_, relative to whole‐network resistance. Adaptation was simulated in networks having capillaries of different radii (*x*‐axis) and while requiring either a high‐ (Panel A) or a low‐ (Panel B) capillary flow velocity. High flow velocity through narrow capillaries results in high *P*_in_ and low VFR (panel A, upper and middle curve, left side). In this case much of the network resistance will reside in the capillaries (lower curve, left), something that cannot be changed by structural changes in the upstream arteriolar network. Note that the low VFR is not caused by low *ψ*_habitual_; in all cases all noncapillary vessels eventually reaches *ψ*_habitual_ = 0.5.

In case of a low capillary flow velocity (Panel B), only a low *P*_in_ is required even for narrow capillaries (left side of the curves). VFR will be larger (compared to similar sized capillaries of Panel A) since a larger fraction of the network resistance will reside outside the capillary bed (lower curve).

Moving to the right in both panels, the pressure needed to drive the blood through the system declines and so does the relative resistance of the capillary bed. As expected this is associated with a larger VFR. Note that this is not in conflict with the results shown in Figure 3 since all data are now for the same value of the endothelial function *γ*. A lower value of *γ* would in all cases result in an increase in *P*_in_ and the other way around. Note also that over a range of capillary radii (and flow velocities) *P*_in_ is quite uniform due to variable adaptation of the surrounding network (in particular upstream), while the resulting VFR varies substantially.

## Discussion

**Figure 6:**
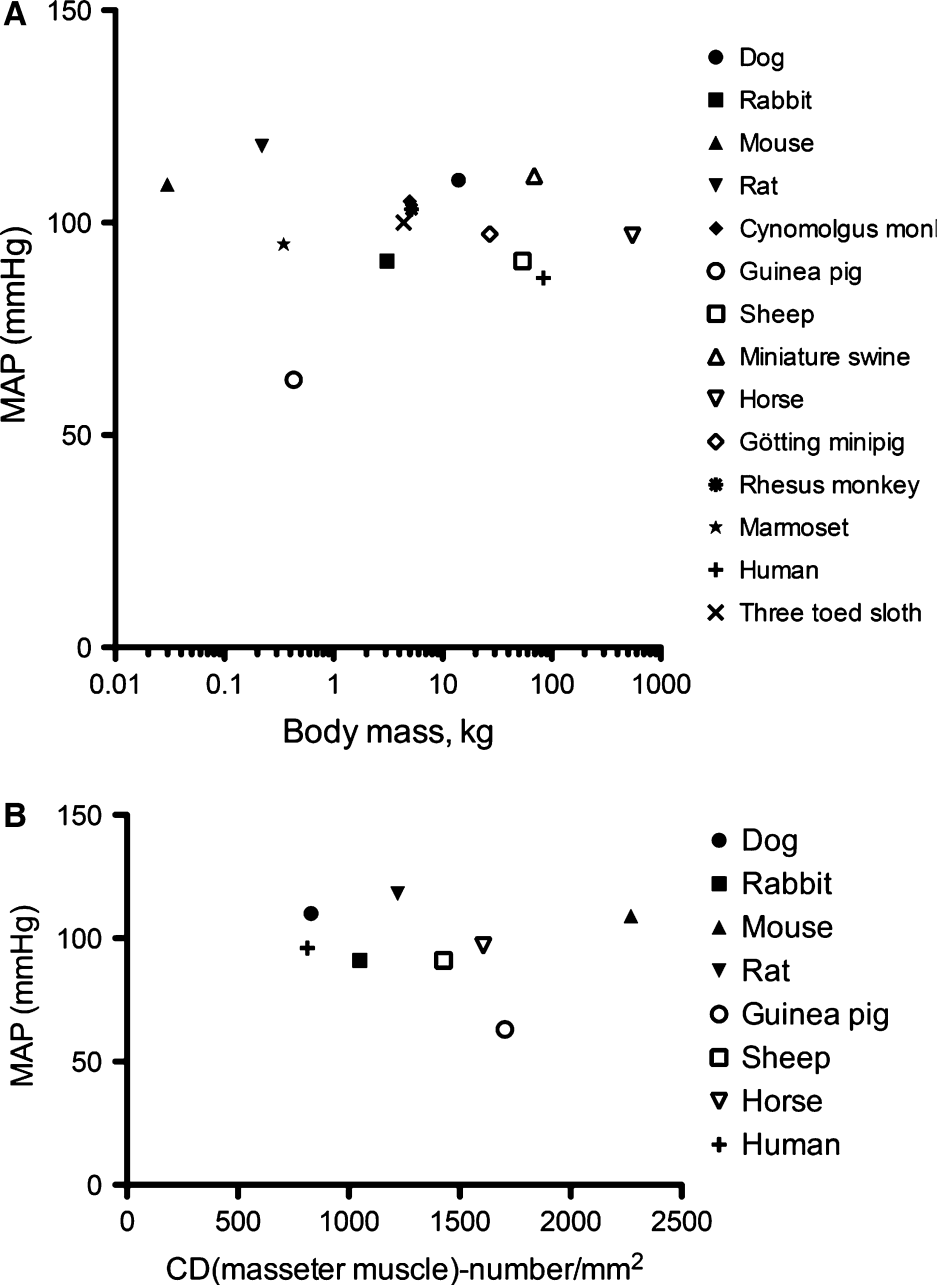
Panel (A): Mean arterial pressure as a function of body mass in 14 different species of mammals. To avoid confounders (e.g., anesthesia, stress, etc.) data are included only from experiments where mean arterial pressure (MAP) has been measured telemetrically in chronically instrumented, conscious, and nonstressed animals. Species included are: dog (Haushalter et al. 2008), rat (Sithisarn et al. 2013), rabbit (Guild et al. 2012), guinea pig (Hess et al. 2007), mouse (Kim et al. 2008), cynomolgus monkey (Haushalter et al. 2008), sheep (King et al. 2007), Yucatan miniature swine (Myrie et al. 2012), Göttingen minipig (Stubhan et al. 2008), Rhesus monkey (Regan et al. 2009), marmoset (Wood et al. 2005), horse (body mass estimated) (Hornicke et al. 1977), three‐toed sloth (Duarte et al. 2003) and human (Damkjaer et al. 2014). Human data are not telemetric. Panel (B): Relation between mean arterial pressure (MAP) and capillary density (CD) in the masseter muscle of eight different species. Data points are mean values from references (Kayar et al. 1988) (horse), (Stal et al. 1996) (human), and (Schmidt‐Nielsen and Pennycuik 1961) (all other species). The figure only includes the species from Panel (A) for which telemetric mean arterial pressure (MAP) data are available (except for humans).

The main result of this study is the demonstration of a relation between VFR and *P*_in_. This relationship may explain why “high performers” such as mammals have a high VFR and a high MAP (Fig. 3). We assume that *P*_in_ is a proxy for MAP, as the majority of the peripheral resistance is found in the microcirculation (Pries et al. 1995a). The results further show that growth and remodeling in the individual noncapillary vessel may underlie the ability of the network to maintain homeostasis alongside with changes in *P*_in_ (Fig. 2) (Jacobsen et al. 2008; VanBavel and Tuna 2014). They also show the ability of the network to deliver a specified capillary flow, while preserving the necessary VFR. Apart from a high pressure, high VFR is associated with a low endothelial function (Fig. 3) and with structural adaptation to intermediate levels of basal activation of the SMC. This activation level is also the most appropriate regarding efficient short‐term flow control since it allows for both up‐ and downregulation of network flow (Fig. 4). Finally, design of the capillary bed is inherently restricted by a trade‐off between efficiency of the exchange process on one side and preserving a high VFR on the other side (Fig. 5). The optimum in this regard is likely to underlie the relatively invariant size of the capillary explaining why MAP, apart from being high, is also uniform across mammals of vastly different size (Fig. 6, panel A). Although MAP is remarkably independent of body size within a class (Seymour and Blaylock 2000; Enok et al. 2014), it varies substantially between classes (Hillman and Hedrick 2015; Schmidt‐Nielsen and Pennycuik 1961; Seymour and Blaylock 2000). To that end it is noteworthy that systemic pressure greatly exceeds the pulmonary ditto in species with anatomical or functional separation between pulmonary and systemic circulations (Wang et al. 2003; Jensen et al. 2014). In the absence of such separation, the systemic pressure must remain low to allow for a sufficiently thin lung diffusion barrier while still avoiding edema formation (Burggren et al. 2014; Jensen et al. 2014; Hillman and Hedrick 2015). This observation, however, does not identify the benefits per se of having a high systemic pressure; that is, why does it evolve despite a disadvantageous larger cardiac workload? (Jensen et al. 2014). The answer indicated by the present results is that only a high‐pressure circulation can meet tissue demand at all levels of metabolic activity while occupying only a fraction of total tissue volume.

It is important to recognize that *P*_in_ and network structure, and hence network resistance, are interdependent entities that evolve until homeostasis, that is, the habitual state, is established. Our suggestion that the need for a certain VFR could drive the system toward a specific state (Fig. 3) should be perceived as being a long‐term evolutionary consequence of selection, rather than based in some adaptive physiological mechanism per se. Although this overall level of VFR, similar to other physiological characteristics, is likely to be specific for, for example, mammals, it may well change in the individual organism as a function of age, training condition, etc.

The present model is a highly simplified description of the real system. We have previously addressed many of these simplifications (Jacobsen et al. 2003, 2008, 2010) and recently reviewed aspects of the numerous microvascular networks models published in recent years (Jacobsen et al. 2009). In particular the influence from wall shear stress has appeared in different formulations. Pries et al. (1995a) noted a relation between local shear‐stress level and local pressure in the network and this relation was incorporated into later models (see e.g., (Pries et al. 1998)). We suggested a formulation (Jacobsen et al. 2003) where the endothelial signal is “diluted” in the wall; hence the rationale outlined in the model section and Appendix D, where influence from shear stress depends on the amount of wall material relative to the endothelial surface. Pries et al. (2005) suggested a similar formulation but where influence from wall thickness was measured relative to a reference value, rather than relative to endothelial surface area. A link to circumferential wall stress is present in both formulations, since changes in circumferential stress may cause a change in the amount of wall material.

Regarding the habitual stress level maintained in each vessel (Jacobsen and Holstein‐Rathlou 2012), the formulation of Appendix F reflects distribution of wall:lumen ratios (anatomical) and pressures (physiological) in the microcirculation (Gore 1974; Pries et al. 1995a, 2001). This simple descriptive formulation was chosen due to lack of knowledge as to what causes the abovementioned anatomical features. Although a similar stress distribution could be modeled by systematic variation in wall material constants through the network it would not change the basic problem, that the cause of the observed anatomical distribution is unknown.

The model relies on growth and remodeling in the individual noncapillary vessel without a priori setting of target values for shear stress or network inlet pressure (Jacobsen et al. 2003, 2010). Although a stable network inlet pressure results from a given simulation, its exact value will depend on the specific parameters in each simulation and it is therefore not directly comparable to MAP in the intact organism. Rather, the overall question is how *P*_in_ relates to other central factors, such as VFR and *γ*. In the following we will discuss some of the central assumptions and limitations of the model.

### Structural adaptation of noncapillary vessels

An infinite number of combinations of different vessel morphologies and different *P*_in_ can give a desired capillary flow. It is therefore not trivial why MAP appears so relatively constant within mammals. Some “force” must pull the system toward a solution space of limited size. To that end we assume that increasing the size of a vessel comes with a “cost” reflected in the level of shear stress necessary to sustain a certain vessel size. This “cost” increases with vessel radius and with relative wall thickness in a greater than linear fashion (se Eq. A11 Appendix D). All else being equal, all noncapillary vessels in the network will therefore have preponderance toward shrinking rather than expanding. This effect is opposed by the rise in *P*_in_ and wall shear stress that follows when the same flow passes through more narrow vessels. Consequently, the system will settle at the point where all noncapillary vessels are as small and thin‐walled as possible, but still meets the homeostatic requirement of the vascular wall (i.e., that $\frac{\overset{¯}{S}}{{\overset{¯}{S}}_{\mathrm{habitual}}}=\frac{\overset{¯}{\psi }}{{\overset{¯}{\psi }}_{\mathrm{habitual}}}=\frac{c_{\mathrm{wall}}}{c_{\mathrm{wall},\mathrm{habitual}}}=1$) as well as the capillary flow requirement. Since relative wall thickness reflects local transmural pressure, the consequence is that the necessary capillary flow can be achieved more “cheaply”, the lower the transmural pressure. Thus, even though the pressure‐volume work of the heart does not enter the equations of the present model, the energetically favorable situation of a lower, as compared to a higher MAP, is reflected indirectly through Eq. A11.

### Properties of the capillary bed

We assume that the efficiency of the diffusion process between the capillary and the metabolically active tissue must be central in shaping a circulatory system. The diffusion process must be relatively invariant across tissues and species, and is associated with a certain average capillary flow‐velocity that matches tissue metabolic activity. In the most basic, approximated view, the demand of the Krogh cylinder (Krogh 1919) of tissue surrounding the capillary must be met. To that end capillaries must have a certain minimum diameter that allows for passage of blood cells, a length suitable for the exchange process (in many cases also reflecting parenchymal structure) and a certain density (i.e., intercapillary distance). Between animals (Schmidt‐Nielsen and Pennycuik 1961; Wiedeman 1963) as well as between tissues, there is considerable variation in these parameters. Perfusion patterns of the microcirculation in vivo also show both spatial and temporal heterogeneity (Pries et al. 1995b). Over time, however, variation in perfusion must average out, giving an average flow velocity that ensures sufficient perfusion. Consequently, we use flow‐velocity in capillaries of a given size as a proxy of whether tissue demand is met or not.

That capillary flow velocity is likely to be a regulated parameter become evident when comparing large and small animals. Between mammalian species basal metabolic rates (BMR) scale with body mass in a power‐law manner. Hence, smaller animals have a higher metabolism per unit body mass (White and Seymour 2003). Interestingly, capillary density tends to be higher in small animals and hence scale positively with BMR, although also influenced by other factors (Schmidt‐Nielsen and Pennycuik 1961). Consequently, MAP tends to be uniform across a broad span of capillary densities (Fig. 6B). This indicate that the higher BMR in small animals cannot be sustained alone by increasing capillary flow‐velocity; an increase in capillary density is also needed, most likely because at some point, transport to the tissue cells become restricted by diffusion rather than by flow through the individual capillary.

The present model has no specific scale as regards the capillary density; it is assumed that density corresponds to (i.e., is adjusted to) tissue demand, such that the latter is met on average, for the given capillary flow‐velocity in capillaries of a given size. As shown in Figure 5, a primary conclusion from the simulations is that *P*_in_ is quite insensitive to the exact value chosen for capillary flow‐velocity. The same holds for capillary radius. This insensitivity stems from the compensatory structural adjustment of the network upstream of the capillary bed. Such adjustment is possible as long as this upstream part of the network holds the majority of the network resistance and, as an important consequence, also provides the network with a high VFR. Therefore, we would anticipate that animals with wide variations in capillary density (reflecting variation in BMR), and even under some variation in average capillary flow velocity and radius, would end up with a similar *P*_in_ at a given, comparable position in their circulatory system. Furthermore, large animals, as compared to small, do not need a large number of additional bifurcations in their vascular system to cover the much longer distances in their body; the low resistance, conductance vessels are much longer in large animals scaling with body mass in a power‐law manner (Holt et al. 1981). At the level of the resistance vasculature and capillary bed, network structure is therefore likely to be very similar across the different species, since in all cases this part of the vasculature must supply the surrounding tissue (West et al. 1997). Although larger species have more microvascular units, these are placed in parallel and a fraction of the circulation of a large species is therefore in a sense comparable to the total circulation of a smaller species. Hence,we argue that *P*_in_ found at the entrance to the microcirculation should not differ much between the species of a class. Indeed, this could be a main reason that MAP does not differ much either. Note that as mentioned previously, in the present simulations no attempt is made to set parameters in such a way as to reach the MAP actually found in mammals. Using the standard parameter setting of the present simulations, however, (see e.g., Fig. 2) the final network inlet pressure (here approx. 7 kPa ≈ 50 mm Hg) is realistic for the position in network that correspond to the final vessel size (diameter of 20–25 *μ*m of the most upstream arteriole) when comparing to literature data (Pries et al. 1995a).

Figure 5 illustrates the trade‐off in the design of the capillary bed. To optimize the diffusion‐driven exchange between blood and tissue the capillary should be as narrow as possible, but this comes with a price. Network resistance, and hence *P*_in_, increases and VFR is reduced, since a larger fraction of the resistance in that case resides in the capillary bed. These effects are more pronounced for large flow velocities which, on the other hand, allows for a larger overall perfusion. In contrast, very large capillaries would, for a similar overall perfusion, reduce the efficiency of the exchange process but would allow for a low *P*_in_ and a large VFR. Collectively one would therefore expect an optimum somewhere in between. Yet, as shown in Figure 5 for a given *P*_in_ there is room for substantial variation in capillary size and flow velocity but only at the expense of variation in VFR. It should be noted that in vivo some capillaries may be nonperfused at rest and only be recruited into carrying flow during an increase in tissue metabolism; this aspect was not considered in the present model (Hudlicka et al. 1982).

### Adaptation of *P*_in_

In the present formulation when the tissue is either over‐ or under‐perfused, it results in adjustments of *P*_in_. No assumption was made as to the physiological mechanisms driving the change in *P*_in_. Potentially, it could involve the well‐known mechanisms behind pressure driven volume regulation in the intact organism (Guyton 1987; Bie et al. 2004). Tissue underperfusion may cause activation of the sympathetic nervous system, increased cardiac contractility, renal retention of salt and water, extracellular volume expansion and a subsequent increase in pressure (Guyton 1987; Bie et al. 2004). While this mechanism is relevant for modern mammals, its involvement in determining arterial pressure on evolutionary timescales, however, remains speculative.

In conclusion, the present model shows that a relation may exist between network inlet pressure and vascular flow reserve. “High performers” such a mammals and birds require a high VFR, but this comes with the price of a high network inlet pressure.

## Figures and Tables

**Figure A1:**
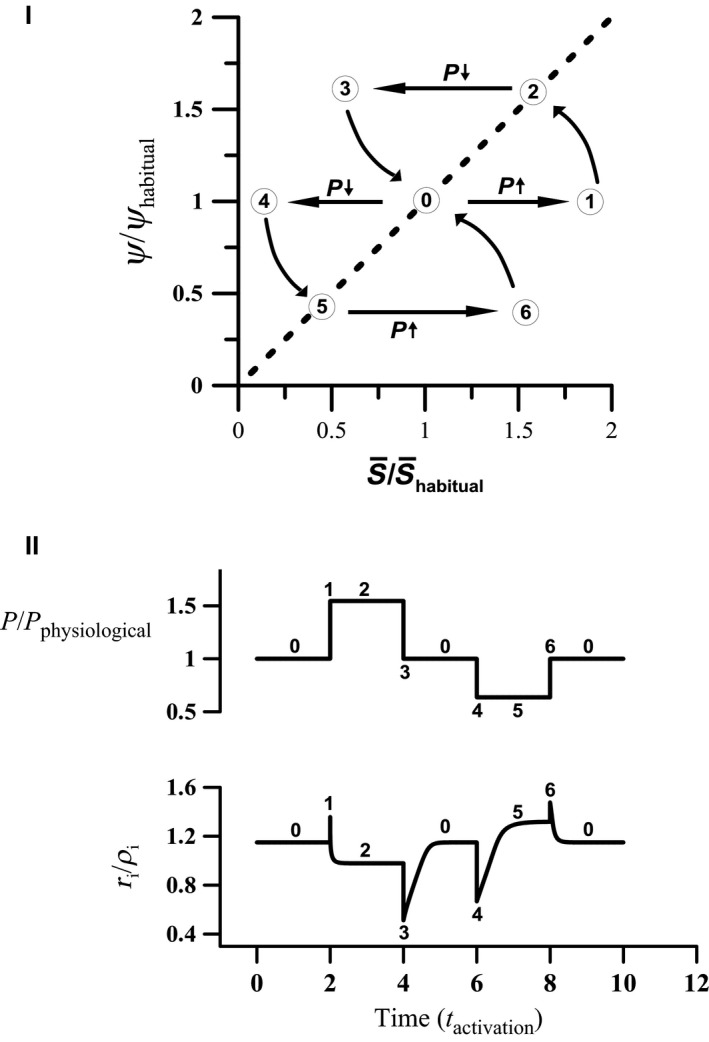
Properties of the myogenic mechanism and remodeling responses. Panel I: Equilibrium line (dashed) for the relation between normalized wall stress and normalized activation. Arrows and numbers indicate the reactions to pressure perturbations of the system shown to the right. Panel II, upper curve: The pressure changes imposed to the system in Panel I and, Panel II, lower curve**,** the subsequent dynamic radius response. Numbers corresponds to those in Panel I. Please see text for details.

**Figure A2:**
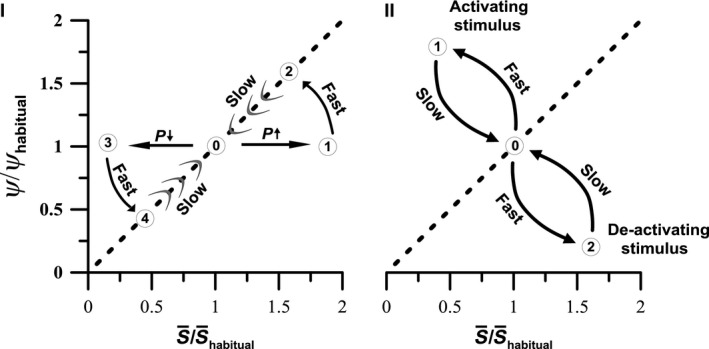
Remodeling and growth responses in noncapillary vessels. Panel I: Remodeling in response a step increase or decrease in pressure and, Panel II, in response to additional activating or deactivating stimuli (other than pressure, please see text for details).

## Appendix

Constants relating to equations of the Appendix are listed in the Appendix Table.

**Table:**

Variable	Value and unit Pascal (Pa), meters (m), seconds (sec)
Parameters	
*C*_*1*_	13500 (Pa)
*C*_*2*_	40 (Pa)
*α*_1_	1.9 (no unit)
*α*_2_	6.7 (no unit)
*b*	100.000 (Pa)
*f*	0.5 (no unit)
*g*	0.7 (no unit)
*k*	100.000 (Pa)
*β*	500 (no unit)
*μ*	0.004 Pa × sec

## Appendix A: Model of the Vascular Wall

A detailed description of the wall model is given in (Jacobsen et al. 2008). In brief, idealized circumferential wall stress, *σ*, can be expressed as (Feldberg et al. 1995):

(A1) $$\sigma =\sigma_{e}+\sigma_{a}$$

where *e* and *a* refer to isotropic, parallel elastic and active muscular components, respectively. The elastic part is modeled as consisting of two components (e.g., collagen and elastin):

(A2) $$\sigma_{e}=C_{1}\left(e^{\alpha_{2}\epsilon }-1\right)+C_{2}\left(e^{\alpha_{2}\epsilon }-1\right)$$

where *ε* = (*L*/*L*_0_ – 1) is the strain and *L*_0_ denotes tissue‐length at zero transmural pressure. The active component is modeled as a smooth, triangular function (Feldberg et al. 1995; Jacobsen et al. 2008):

(A3) $$\sigma_{a}=be^{\left[-{\left(\left(\varepsilon -f\right)/g\right)}^{2}\right]}$$

where *b* determines the maximum active stress the wall can develop and *f* and *g* determines the position and width, respectively, of the active stress distension curves (Feldberg et al. 1995; Jacobsen et al. 2008).

With *ρ* being the radius of a layer within the wall when transmural pressure and activation are both zero and *r* being the radius of the same layer at a certain level of pressure and activation, wall area conservation can be expressed as: $r^{2}-r_{i}^{2}=\rho^{2}-\rho_{i}^{2}$, where *i* refer to the inner radius of the vessel wall.

With *o* referring to outer radius of the vessel wall the transmural pressure, *P* is given by Laplace's law:

(A4) $$P=\int_{r_{i}}^{r_{o}}\frac{S}{r_{i}}dr=\int_{1}^{\eta }\frac{\sigma_{e}}{r_{\rho }}dz+\psi \int_{1}^{\eta }\frac{\sigma_{a}|\psi =1}{r_{\rho }}dz$$

where *S* = *σ*(1 + ε) is the Cauchy stress, *r*_*ρ*_ = *r*_*i*_/*ρ*_*i*_ is the normalized internal radius, $\eta =\frac{\rho_{0}}{\rho_{i}}$ is the relative thickness of the relaxed wall, $Z=\frac{\rho }{\rho_{i}}$ is an integration variable and where the strain is expressed as:$\;\;\varepsilon =\left(1/z\right)\sqrt{r_{\rho }^{2}-1+|z^{2}}-1$. The dimensionless parameter *ψ* expresses the degree of SMC activation, normalized to lie between 0 and 1.

Finally, the expression for the average circumferential wall stress at a given SMC‐activation and transmural pressure takes the form:

(A5) $$\begin{array}{cc} \overset{¯}{S}=\frac{{\mathrm{Pr}}_{i}}{r_{0}-r_{i}} & =\frac{r_{\rho }}{\left(\sqrt{\eta^{2}-1+r_{\rho }^{2}}\right)-r_{\rho }}\\ & \;\left(\int_{1}^{\eta }\frac{\sigma_{e}}{r_{\rho }}dz+\psi \int_{1}^{\eta }\frac{\sigma_{a}|\psi =1}{r_{\rho }}dz\right) \end{array}$$

## Appendix B: Smooth Muscle Cell Activation

The pseudo steady‐state activation, *ψ*_pss_ of a vessel in a specific state (i.e., at a given radius, transmural pressure and flow) is determined by the sum influence from the different vasomotor mechanisms, *x:*

(A6) $$\psi_{\mathrm{pss}}=\sum_{x}\psi_{\mathrm{pss}},x$$

Since time is needed to change the contractile state of the SMC, the actual activation, *ψ* does not reach *ψ*_pss_ instantaneously, but rather approaches it at a certain rate. This process is modeled as a simple first order process (Jacobsen et al. 2008):

(A7) $$\frac{d\psi }{dt}=\frac{1}{t_{\mathrm{activation}}}\left(\psi_{pss}-\psi \right)$$

When the system is at steady state, that is, when there is no more movement of the vascular wall, the outwardly directed force caused by the intravascular pressure is balanced by the inwardly directed force generated by the wall. Any disturbance of the system induces force disequilibrium, followed by movement of the wall. The new vessel configuration causes a shift in the contributions to *ψ*_pss_ from the different vasomotor mechanisms which in turn causes a shift in *ψ* and a concomitant adjustment of vessel radius. This again changes the input to *ψ*_pss_ and, in case the stimulus is maintained, adjustment proceeds until a new equilibrium is established.

## Appendix C: Regulation of SMC‐Activation I: The Myogenic Response

Smooth muscle cells of the vascular wall are sensitive to the intravascular pressure level. Most likely this is sensed through changes in circumferential wall stress (Davis and Hill 1999). In the absence of other factors influencing smooth muscle cell activation (corresponding to an isolated, endothelium free vessel in a pressure myograph), stress‐dependent activation of the wall SMC establishes the characteristic myogenic relation between intravascular pressure and radius (Jacobsen et al. 2008). In microvascular networks there appear to be a characteristic gradient in myogenic responsiveness (Davis 1993). Vessels tend to exhibit myogenic reactivity over a range of pressure centered on their normal physiological pressure. In the present formulation this will correspond to the point at which circumferential wall stress is identical to the normal physiological stress of the wall, ${\overset{¯}{S}}_{\mathrm{habitual}}$ These features are modeled as previously (Jacobsen et al. 2010) as follows:

(A8) $$\psi_{\mathrm{pss},\mathrm{myogenic}}=\psi_{\mathrm{habitual}}{\left[\frac{\overset{¯}{S}}{{\overset{¯}{S}}_{\mathrm{habitual}}}\right]}^{f\left({\overset{¯}{S}}_{\mathrm{habitual}}\right)}$$

where:

(A9) $$f\left({\overset{¯}{S}}_{\mathrm{habitual}}\right)=2-\left[\frac{{\overset{¯}{S}}_{\mathrm{habitual}}}{k}\right]$$

Figure A1 panel I shows schematically how the myogenic activation changes as a function of circumferential stress, with both variables given relative to their habitual values. The figure shows the excursions in relative activation and relative stress following a series of pressure changes (upper curve of Panel II). The corresponding changes in relative radius are shown in the lower curve of Panel II. The excursions follow the directions of the arrows 0 → 1 → 2 → 3 → 0 and on to 4 → 5 → 6 and back to 0. The corresponding numbers are indicated on the two curves to the right. At the habitual pressure and considering only to myogenic mechanism, the vessel will remain at the point (0) where:

(A10) $$\frac{\overset{¯}{S}}{{\overset{¯}{S}}_{\mathrm{habitual}}}=\frac{\psi }{\psi_{\mathrm{habitual}}}=\frac{c_{\mathrm{wall}}}{c_{\mathrm{wall},\;\;\mathrm{habitual}}}=1$$

Following a step increase in pressure, circumferential stress increases sharply (horizontal arrow to the right from 0 → 1) and the vessel dilates acutely due to force disequilibrium between the wall and the luminal pressure. Due to the increased stress stimulus activation increases, (curved, ascending arrow from 1 → 2), allowing the vessel to simultaneously reduce its diameter and causing a concomitant reduction in stress. Eventually when the equilibrium line is reached at 2, the radius has settled at a new lower value, but with the vessel wall continuously experiencing a higher stress and hence higher activation as compared to the initial state at 0.

If pressure is now reduced by the same amount as it was initially raised (going from 2 → 3) the system starts from a state of high activation and the vessel will once again experience force disequilibrium. This results in a radius overshoot, but now in the opposite direction (a collapse). As activation hereafter declines the vessel dilates and wall stress increases until equilibrium is reestablished (curved arrow from 3 and back to 0). The same process, but in the opposite direction is given by the excursion 0 → 4 → 5 → 6 and back to 0.

Myogenic reactivity is a property intrinsic to the vascular SMC. It is considered to provide the vessel with a certain level of tone upon which other vasomotor mechanisms can operate (Davis and Hill 1999). Its input to the activation function can therefore vary between 0 (when transmural pressure is zero) and 1 at a sufficiently high transmural pressure (and at all pressure levels above). Hence, we assume the myogenic response cannot give a negative input to the total activation.

## Appendix D: Regulation of SMC‐Activation II: The Shear‐Stress‐Sensitive Mechanism – Influence from the Endothelium

Through a variety of mechanisms the vascular endothelium has a strong influence on the activation state of the vascular wall. Among these, wall shear stress is known as a central factor in acute diameter regulation in the microcirculation. Generally, an increase in the local shear stress, *τ* causes SMC relaxation and vasodilatation (Koller et al. 1993). Experimental studies have shown that a sustained change in wall shear stress causes structural remodeling as well, with an increase in *τ* causing outward remodeling and the other way around (Pourageaud and De Mey 1997; Blus et al. 2001). Studies of vessels in organ culture indicate that this remodeling effect indeed operates through changes in SMC‐activation (Pistea et al. 2005). In the present formulation therefore, the effects of *τ* both acutely and chronically, are unfolded through its effects on *ψ*. We make no a priori assumptions as to the homeostatic level of shear stress in any given vessel (Jacobsen et al. 2003). Rather it is assumed that *τ* is a variable that is indirectly regulated in such a way that the influence from the endothelium on the vascular wall remains constant and that structural remodeling enables *τ* to increase or decrease toward a level at which this is fulfilled. For simplicity we consider *c*_wall_ to be the same in all vessels; In vivo*,* however, it may show variation through the network as a function of, for example, vessel size. The influence from the endothelium is therefore formulated as previously (Jacobsen et al. 2003). Given that *c*_wall_ (equilibrium value *c*_wall,habitual_) symbolizes the influence of the endothelium on the wall, it should incorporate the endothelial surface area, *A*, influenced by shear and the underlying volume, *V*, of wall material actually being influenced. Collectively this is formulated as:

(A11) $$c_{\mathrm{wall}}=\frac{A\left[{\left(\tau +1\right)}^{\gamma }-1\right]}{V}$$

where, for a given vessel, *A* = 2*πr*_*i*_ *l* is the endothelial surface area, $V=\pi \rho_{i}^{2}\left(\eta^{2}-1\right)l$ is the wall volume and *γ* modulates the effect of shear stress on the endothelium, that is, represents the “endothelial function”. Eventually the difference between the homeostatic and the actual endothelial influence on the wall, *c*_wall, habitual_‐*c*_wall_ feed into the common SMC‐activation function, hereby influencing vessel radius both acutely and chronically, that is,

(A12) $$\Delta \psi_{\mathrm{endothelial}}=\beta \left(c_{\mathrm{wall},\mathrm{habitual}}-c_{\mathrm{wall}}\right)$$

where *β* is an appropriate relaxation factor determining the rate of convergence.

The above considerations are general in the sense that they do not distinguish between different endothelial factors that influence the wall in response to shear, for example, NO, prostaglandins, current, etc. Although these factors differ in the chain of events leading from shear on the endothelial surface to the final influence on SMC‐activation, they are all subject to the same basic conditions, namely the size of the endothelium and the size of the underlying wall mass. There are indeed experimental indications that it is reasonable to consider the latter two factors. Bakker et al. (2003) measured structure and shear stress along unbranched segments of first‐order rat cremaster arterioles finding that these vessels have a larger radius down‐stream as compared to upstream and that down‐stream shear stress is only 2/3 of the upstream value. At the same time up‐ and downstream wall cross‐sectional areas are approximately the same. Taken together these features may therefore lead to a relatively uniform endothelial influence on the wall along the vessel.

Endothelial shear stress is central in determining luminal vessel radius. The influence from shear stress is seen in both large conductance vessels and in resistance vessels. In the process of remodeling it enables a given vessel to adjust structurally to the amount of flow carried. Vessels forced to carry a larger flow will expand luminally, seen, for example, in the expansion of collaterals under experimental or pathological conditions but also under normo‐physiological conditions such as long‐term physical training (Miyachi et al. 1985; Unthank et al. 1996). A chronically reduced flow will have the opposite effect (Pourageaud and De Mey 1997).

In the present formulation, endothelial shear stress influences local SMC‐activation through Eq. A11 & A12 and hence determines vessel radius both acutely (through changes in tone) and – in turn – chronically (through changes in structural radius). We make no a priori assumptions about the magnitude of the shear stress in a given vessel, only about the degree to which shear stress influence the vascular wall (as modulated by the exponent *γ*). Measurements and network simulations indicate a substantial variation in microvascular wall shear stress with generally higher levels found in arterial‐ as compared to venous side vessels (Pries et al. 1995a). As shown in Figure 2 panel F, the microcirculatory structure emerging from the present simulations indeed gives rise to shear‐stress levels that are in accordance with these findings.

## Appendix E: Response to Tissue Metabolism

Although tissue perfusion is not homogeneous, every part of the tissue must on average over time be perfused adequately. A simple, approximate measure of sufficient tissue perfusion is the flow through any well‐defined tissue region in vessels of capillary size, the latter of which is relatively invariant in the living organism (Wiedeman 1963). In the present formulation where capillary radius is fixed in the individual simulation, it is assumed that tissue metabolic need is satisfied when the blood flows through the capillary bed with a certain velocity. This desired network flow, is thus a measure of tissue metabolism. With the tissue metabolic factor defined as m = (desired network flow – actual network flow)*,* the change in *P*_in_ was implemented as

$$\Delta P_{\mathrm{in}}=\frac{m}{\text{desired network flow}}\times 1\;Pa,\mathrm{when}\;m>0$$

and

(A13) $$\Delta P_{\mathrm{in}}=\frac{m}{\text{actual network flow}}\times 1\;Pa,\mathrm{when}\;m<0$$

*ΔP*_in_ is therefore restricted to the interval −1 to 1 Pa and the steps become larger the larger the deviation between desired and actual flow.

## Appendix F: Habitual Activation

A general feature of vascular networks is the presence of a certain basal SMC‐activation in noncapillary vessels. This basal activation is present under what can be considered the “resting state” of the network where the vessels are exposed to their physiological pressure. It provides the vessels with a certain “tone”, allowing for rapid up‐ or downregulation of local perfusion (Davis and Hill 1999). If the network is maintained in a given state, that is, exposed to a given pressure difference and delivering the necessary flow, then eventually all transients will decay. This happens when all vessels in the network has reached their long‐term homeostatic (i.e., habitual) point. Changing the pressure difference across the network and/or the flow demanded by the tissue, does not change the habitual state per se; but changes vessels morphology which in turn allows for the habitual state to be re‐established.

We assume that the remodeling response per se, induced by changes in activation, is eutrophic in nature; the same amount of wall material is restructured around a lumen of a different size (Mulvany 1999). As previously (Jacobsen et al. 2008) this was formulated as a simple first‐order process with the change in structural internal radius, $\rho_{i}$ being:

(A14) $$\frac{d\rho_{i}}{dt}=\frac{1}{t_{\mathrm{remodeling}}}\left(\psi_{\mathrm{habitual}}-\psi \right)\rho_{i}$$

Hence, if *ψ* > *ψ*_habitual_ structural radius will decrease (Bakker et al. 2002, 2004) whereas if *ψ* < *ψ*_habitual_ it will increase (Pistea et al. 2005) In both cases this process does not in itself cause any changes in the wall cross‐sectional area. *ρ*_*i*_ is multiplied on the right side of the equation to scale *Δρ*_*i*_ to the size of *ρ*_*i*._

### Habitual Circumferential Wall Stress

The average circumferential wall stress, $\overset{¯}{S}$ in the microvascular wall is a function of transmural pressure, internal radius, and wall thickness (c.f. Eq. A5). $\overset{¯}{S}$ is generally found to increase with increasing vessel size on both sides of the capillary bed (Pries et al. 2001). In the present model, the average circumferential wall stress of a given vessel exposed to its physiological pressure level and after all transients have decayed, is denoted, ${\overset{¯}{S}}_{\mathrm{habitual}}$. As for *ψ*_habitual_ it is assumed that ${\overset{¯}{S}}_{\mathrm{habitual}}$ represents a homeostatic point where vascular function in short‐term flow regulation is optimal. As an estimate for ${\overset{¯}{S}}_{\mathrm{habitual}}$ we use a function for $\overset{¯}{S}(r_{i})$ based on literature data (Pries et al. 2001):

(A15) $$\overset{¯}{S}\left(\right.r_{i}=\left(25\times 10^{6}\right)r_{i}^{0.624}$$

where it is presumed that the experimentally measured radius represents the internal radius, that is, the width of the blood cell column. Since *r*_*i*_ changes rapidly with fluctuations in pressure and SMC activation, we use in the present simulations instead the structural inner radius *ρ*_*i*_ that changes much more slowly over time but reflects a comparable relation between vessel size and circumferential wall stress as does Eq. A15:

(A16) $${\overset{¯}{S}}_{\mathrm{habitual}}\left(\rho_{i}\right)=\left(25\times 10^{6}\right)\rho_{i}^{0.624}$$

Following a sustained perturbation, remodeling alone may not be able to bring $\overset{¯}{S}$ back to ${\overset{¯}{S}}_{\mathrm{habitual}}$. In that case it is assumed that the vascular wall exhibits a slow trophic response (hyper‐ or hypotrophic, i.e., a change in the local cross‐sectional area, *A* of the vascular wall) which corresponds to changing the relative wall thickness, *η* at a given *ρ*_*i*_:

(A17) $$\frac{d\eta }{dt}=\frac{1}{t_{\mathrm{trophic}}}\left(\overset{¯}{S}-{\overset{¯}{S}}_{\mathrm{habitual}}\right)$$

Hence, if $\overset{¯}{S}>{\overset{¯}{S}}_{\mathrm{habitual}}$ the wall will increase in relative thickness (absolute and/or relative) and the other way around. Similar to Eq. A14 the right side of Eq. A17 is, in the implementation, scaled appropriately to the size of *η*.

Every noncapillary vessel in the network will now adapt toward the state given by Eq. A10. Appendix Figure A2 panel I, shows schematically the consequences of an isolated, sustained change in pressure (such that permanently $\overset{¯}{S}\neq {\overset{¯}{S}}_{\mathrm{habitual}}$ and $\overset{¯}{\overset{¯}{\psi }}\neq {\overset{¯}{\psi }}_{\mathrm{habitual}}$). In that case remodeling alone may cause simultaneous normalization of stress and activation returning the two variables to their habitual values (Jacobsen et al. 2008). Note that the *y*‐axis is normalized to ${\overset{¯}{S}}_{\mathrm{habitual}}$ and the *x*‐axis is normalized to ${\overset{¯}{S}}_{\mathrm{habitual}}$. Consequently the homeostatic state is represented by a single point (0) in (1, 1) A step change in pressure (represented by horizontal arrows) is followed by myogenic correction of radius (vertical curved arrows) until the pseudo steady‐state line is reached. In turn, if the pressure stimulus is sustained, the slow remodeling response (gray arrows) causes rearrangement of the wall material around a lumen of a different size, which eventually returns the vessel to the point of operation at 0. Although, during this process, there is a change in structural internal radius, the point of operation in (1, 1) does not shift position due to the normalization to ${\overset{¯}{S}}_{\mathrm{habitual}}\left(\rho_{i}\right)$ and to *ψ*_habitual._

Normally, and unlike the situation Appendix Figure A2 panel I, activation of the vascular wall is influenced by several vasomotor mechanisms and not just the myogenic mechanism. This is due to additional requirements such as the need for the network to deliver a certain flow. In that case it may be impossible to reach the point of operation by remodeling alone. This situation is illustrated in Panel II where a stimulus changing the activation causes an acute, oblique movement in the plane. For instance, an increase in metabolism will cause SMC deactivation (fast downward movement) but since vasodilatation is associated with an increase in *S* there will be a simultaneous rightward movement in the plane. Although the metabolic stimulus may cease in parallel with the resulting increase in flow, wall stress cannot in this case be normalized by inward remodeling since that would conflict with the need for an increased perfusion. Since $\overset{¯}{S}>{\overset{¯}{S}}_{\mathrm{habitual}}$ there will, however, be a continuous stimulus for increasing the amount of wall material (a trophic response, c.f. Table 2). As time passes, this process therefore ensures that radius can remain large to permanently eliminate the metabolic stimulus (i.e., the tissue metabolic need is satisfied) while at the same time ensuring normalization of the stress (since the relative wall thickness increases) and therefore also of stress‐induced activation. Hence, the system can, albeit slowly (up‐ and leftward curved arrow), return to the point of operation if a trophic response operates alongside with remodeling.

In case of a metabolic stimulus that causes an increase in activation instead of a decrease (for instance due to high *PO*_2_ associated with overperfusion) the system behaves inversely (arrows above the dashed line).

## Appendix G: Network Hemodynamics

The sum of the flows, *Q* entering and leaving any node equals zero (Kirchhoff's law) so that (Jacobsen et al. 2003):

(A18) $$\sum_{j}Q_{j}^{n}=\sum_{j}C_{j}^{n}\Delta P_{j}^{n}=0$$

where $Q_{j}^{n}$is the flow, $C_{n}^{j}$ is the vascular conductance and $\Delta P_{j}^{n}$is the pressure drop in the $j^{\prime }th$ vessel entering the $n^{\prime }th$ node. The average transmural pressure in the $j^{\prime }th$ vessel connecting the $m^{\prime }th$ and $n^{\prime }th$ nodes is ${\overset{¯}{P}}_{j}=\left(P^{m}+P^{n}\right)/2$ Assuming that the flow is laminar and if nonnewtonian properties of the blood are ignored, the flow obeys Poiseuilles law and the vascular conductance can be calculated as:

(A19) $$C_{j}=\frac{\pi r_{i}^{4}}{8\mu l_{j}}$$

where *μ* is the viscosity of the blood and *l*_*j*_ is the length of the $j^{\prime }th$ vascular segment. Finally the wall shear stress in the $j^{\prime }th$ segment becomes:

(A20) $$\tau =\frac{4\mu Q_{j}}{\pi r_{i}^{3}}=\frac{r_{i}\Delta P_{j}}{2l_{j}}$$

## Appendix H: Program flow and computational methods

The overall program flow is outlined Figure 1. Using the boundary pressures and the initial values of the vessel radii, node pressures are calculated by solving a linear system based on Eqs. A18 and A19. New values are then found for the differential variables *ψ*,* ρ*_*i*_ and *η*. Hereafter updated values of all algebraic variables can be calculated for each vascular segment. First, calculation of the pressure decline, ΔP along each segment and average transmural pressure, $\overset{¯}{P}$, enables calculation of new values for local $\overset{¯}{S}$, *Q, τ*,* ψ*_pss_, *v, m* and *c*_wall_. The active internal radius *r*_*i*_, is the radius where the pressure generated by the vascular wall is in equilibrium with the transmural pressure. Updated values for *r*_*i*_ are found by iteratively solving:

(A21) $$\overset{¯}{P}-P=0$$

where *P* is given by Eq. A4. In between network outlet pressure is maintained constant. Network inlet pressure is free to drift in the direction governed by the difference between the actual and the desired total capillary flow. This process continues until every vessel and node in the network simultaneously fulfills all convergence criteria. Where required, appropriate relaxation factors are applied in the iteration process to ensure smooth convergence. The size of these factors does not affect the final result.
